# Lyn, Lupus, and (B) Lymphocytes, a Lesson on the Critical Balance of Kinase Signaling in Immunity

**DOI:** 10.3389/fimmu.2018.00401

**Published:** 2018-03-01

**Authors:** Erica J. Brodie, Simona Infantino, Michael S. Y. Low, David M. Tarlinton

**Affiliations:** ^1^Department of Immunology and Pathology, Monash University, Melbourne, VIC, Australia; ^2^Department of Medical Biology, University of Melbourne, Parkville, VIC, Australia; ^3^Immunology Division, Walter and Eliza Hall Institute of Medical Research, University of Melbourne, Parkville, VIC, Australia; ^4^Department of Haematology, Monash Health, Monash Hospital, Clayton, VIC, Australia

**Keywords:** Lyn, systemic lupus erythematosus, cell signaling, SFK, B cell, B-cell receptor, autoimmunity, lupus

## Abstract

Systemic lupus erythematosus (SLE) is a progressive autoimmune disease characterized by increased sensitivity to self-antigens, auto-antibody production, and systemic inflammation. B cells have been implicated in disease progression and as such represent an attractive therapeutic target. Lyn is a Src family tyrosine kinase that plays a major role in regulating signaling pathways within B cells as well as other hematopoietic cells. Its role in initiating negative signaling cascades is especially critical as exemplified by Lyn^−/−^ mice developing an SLE-like disease with plasma cell hyperplasia, underscoring the importance of tightly regulating signaling within B cells. This review highlights recent advances in our understanding of the function of the Src family tyrosine kinase Lyn in B lymphocytes and its contribution to positive and negative signaling pathways that are dysregulated in autoimmunity.

## Systemic Lupus Erythematosus (SLE)

Systemic lupus erythematosus is a heterogeneous autoimmune disease with multiple clinical manifestations including auto-antibodies, dermatological rashes and ulcers, inflammatory markers, hematological deficiencies, arthritis, renal dysfunction, and neurological disorders (1). The incidence of SLE is approximately 1:1,500; however, the prevalence varies significantly with gender, ethnicity, and age (2). The progression of SLE symptoms is mediated by a dysregulation of innate (e.g., dendritic cells, mast cells, and macrophages) and adaptive immune cells (i.e., B and T lymphocytes) (3). Accumulating evidence emphasizes the contribution of B cells in mediating the development of autoimmunity, particularly through the breakdown in tolerance to self-antigens, the secretion of inflammatory cytokines, and the generation of auto-reactive antibodies that result in immune complex deposition in organs such as the kidneys (4–6). In keeping with this, numerous mutations affecting tyrosine kinases have been implicated in autoimmune disease progression (7), highlighting a critical requirement for the stringent regulation of intracellular signaling cascades in immune cells.

This review will focus on intracellular signaling within B cells, specifically on the role of Lyn in regulating these pathways and its contribution to the progression of autoimmune disease.

## Lyn: Src Family Tyrosine Kinase

Lyn (Lck/yes-related novel tyrosine kinase) is a Src family, non-receptor tyrosine kinase found predominantly in myeloid cells and B lymphocytes (8), but it is also detectable in cell types outside of the hematopoietic compartment (9). *Lyn* is located on chromosome 4 A1 in mice and 8q12 in humans (9). In mice, an additional *lyn* gene encoding exons 1–10 is present within the genome but is not transcribed (10). Alternate splicing of exon 2 results in the translation of two different isoforms of Lyn, annotated as Lyn A (56 kDa) and Lyn B (53 kDa), that differ by an insertion within the N-terminal variable domain (11, 12). Functional analysis of the individual isoforms indicates a similar capacity to phosphorylate substrate proteins (12–14); however, both isoforms are required for normal activation and regulation of internal signaling (13, 14).

### Structural/Functional Regulation of Lyn

Lyn shares architectural and sequence homology with the other SFK members present in hematopoietic cells (e.g., Src, Fyn, Yes, Blk, and Hck) (15). The conserved domain organization of SFK members includes an N-terminal/unique domain, Src homology (SH) 3, SH2 and the catalytic/kinase domain. The N-terminal unique domain (SH4) contains sites for myristoylation and palmitoylation that promote localization and interaction with the cellular membrane and also integration into lipid rafts (16–18). Downstream of the N-terminus, SH3 and SH2 domains regulate the conformational and functional state of Lyn (19). Phosphorylation of the inhibitory tyrosine (Y529 in murine Src, Y508 in Lyn) on the C-terminus leads to a closed and inactive conformation via the binding and “latching” of the SH2 domain, which is further stabilized by the interaction with the SH3 domain (20, 21).

Dephosphorylation of the C-terminal tyrosine by phosphatases or competitive binding for the SH2 domain by an interacting protein relieves the inhibitory conformation imposed by SH3/2 domains, which then promotes phosphorylation of the activating tyrosine (Y416 in Src, Y397 in Lyn) within the kinase domain (19, 21, 22). Phosphorylation of Y397, either in *cis* or in *trans*, changes the conformation of the activation loop, permitting substrate binding and kinase activity (19, 22, 23). Kinase activity is mediated by interactions between the ATP binding loop (G loop) and the ATP hydrolyzing loop (catalytic loop) that facilitate optimal positioning and hydrolysis of the λ-phosphate group from ATP, respectively (20, 24, 25). The λ-phosphate group is then transferred to a protein substrate that is positioned adjacent to the catalytic loop (25). Dephosphorylation of the activating tyrosine or phosphorylation of the inhibitory tyrosine by Csk (c-Src kinase) decreases the kinase activity of Lyn and other SFKs (26).

## Lyn Regulates Positive and Negative Pathways in B Cells

Activation of Lyn relies on membrane-bound receptor-type phosphatases such as CD45 and CD148 to dephosphorylate the C-terminal inhibitory tyrosine (27–29). After activation, Lyn binds and phosphorylates substrate proteins that possess tyrosine residues flanked predominantly by acidic residues (30–32). The affinity for particular substrates is further regulated by the phosphorylation of the SH2 domain (33). Key substrates of Lyn include proximal membrane-bound cellular surface receptors containing immunoreceptor tyrosine activating motifs (ITAMs) or immunoreceptor tyrosine inhibitory motifs (ITIMs) within their cytoplasmic tails (34, 35). Phosphorylation of these ITAMs and ITIMs leads to the recruitment and activation of other kinases, phosphatases, and adaptor proteins that enhance or inhibit downstream signaling.

### Positive Signaling Cascade

**Figure 1:**
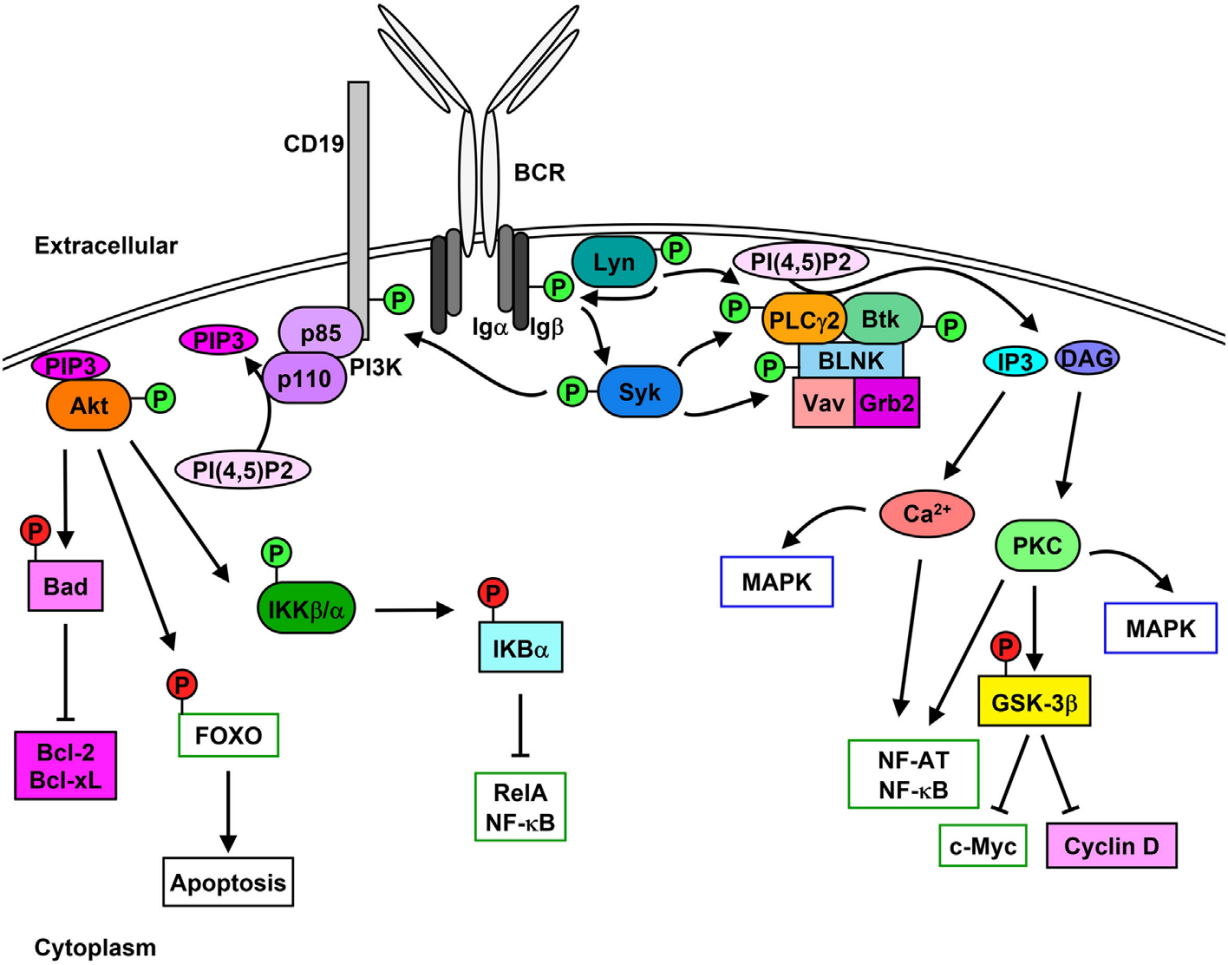
Lyn initiates signaling cascades following B cell receptor (BCR) cross-linking. Stimulation of the BCR leads to the activation [green (P)] of Lyn and Syk which results in Igα/β phosphorylation. PLCγ2 activity leads to the hydrolysis of PI(4,5)P2 to IP3 and diacylglycerol (DAG), which stimulates Ca^2+^ mobilization as well as protein kinase C (PKC) and MAPK activation and cell-cycle progression. Phosphorylation of CD19 leads to the membrane recruitment of phosphatidylinositol 3-kinase (PI3K) and activation of Akt, which phosphorylates downstream proteins leading to their inhibition [red (P)] and up-regulation of pro-survival signaling.

The B cell receptor (BCR) comprises the membrane-bound immunoglobulin (Ig) and the heterodimeric signaling subunit Ig-α/Ig-β (CD79α/β) that contain ITAMs within their cytoplasmic tails (36). Antigen binding to the BCR induces a variety of signaling cascades that are initiated by the proximal kinase Lyn (Figure 1), which phosphorylates Ig-α/Ig-β ITAMs thereby creating docking sites for the recruitment and activation of Syk (37–39). Activated Syk leads to the phosphorylation and activation of downstream molecules such as the adaptor protein SH2 domain-containing leukocyte protein of 65 kDa (SLP-65) (also known as BLNK or BASH), Btk, and PLCγ2 (40). Upon phosphorylation, SLP-65 organizes a signalosome that promotes calcium (Ca^2+^) flux and the differentiation of developing B cells (41). Phosphorylated SLP-65 also allows the recruitment of Bruton’s tyrosine kinase (Btk), Vav Guanine Nucleotide Exchange Factor 1 (Vav1), and Growth Factor Receptor Bound Protein 2 (Grb2) (42).

Phosphorylation of the non-T-cell activation linker (NTAL) by Lyn leads to the recruitment of Grb2, murine son of sevenless homolog (mSOS), and GRB2-Associated Binding Protein 1 (Gab1) (43). Phosphorylation of Gab1 promotes complex formation between SH2 Domain-Containing Transforming Protein 1 (Shc), non-receptor SH2-containing tyrosine phosphatase 2 (SHP-2), and p85 subunit of phosphatidylinositol 3-kinase (PI3K) (44). Syk-mediated phosphorylation of Shc promotes the interaction between Grb2 and mSOS, which activates Ras-MAPK pathways (45), while interaction between Shc and Gab1 leads to the activation of PI3K pathway (46, 47).

Syk-mediated phosphorylation of CD19 enables the recruitment and activation of p85, which also promotes membrane localization of PI3K (48, 49). Syk also phosphorylates E3 ubiquitin-protein ligase Cbl, which permits the interaction with p85 and activation of PI3K (50). Similarly, Syk and Btk phosphorylate B cell phosphoinositide 3-kinase adapter protein 1 (BCAP), which localizes p85 to glycolipid-enriched microdomains (GEMs) after anti-IgM stimulation (51). PI3K catalyzes the generation of phosphatidyl inositol 3,4,5-trisphosphate (PIP3) from phosphatidyl inositol 4,5-trisphosphate (PI(4,5)P2), which in turn is used as a docking site to activate other effector proteins (52). PIP3 is essential for plasma membrane translocation of Akt, placing it in proximity to 3-phosphoinositide-dependent protein kinase 1 (PDK1) and rapamycin-insensitive companion of mammalian target of rapamycin (Rictor)/mechanistic target of rapamycin (mTOR2) allowing threonine and serine phosphorylation of Akt, respectively (53, 54). Akt phosphorylation and activation following BCR cross-linking leads to the inhibition of the pro-apoptotic protein Bad and activation of the pro-survival proteins Bcl-2 and Bcl-XL (55). Additionally, Akt phosphorylates the transcription factor Forkhead box protein O (FOXO) thereby inducing its exclusion from the nucleus and preventing apoptosis while promoting cell proliferation (56, 57). Akt also activates alpha and beta subunits of the IκB kinase (IKKα/β) which phosphorylates IκBα and the p65 nuclear factor kappaB (NF-κB) subunit/RelA (58). IκBα phosphorylation leads to its proteasome-mediated degradation and exposure of the nuclear-localization sequence of RelA, which leads to its translocation and up-regulation of pro-proliferation and pro-survival proteins such as c-Myc and Bcl-2 family proteins (59, 60).

Lyn, Syk, Btk, and Blk can also phosphorylate and enhance the activation of phospholipase C gamma 2 (PLCγ2), which hydrolyzes PI(4,5)P2 to create inositol 3,4,5-trisphosphate (IP3) and diacylglycerol (DAG), stimulating Ca^2+^ mobilization and protein kinase C (PKC), respectively (61–64). PLCγ2 phosphorylation also stimulates the activation of MAPK pathways and nuclear location of NF-κB and nuclear factor of activated T cells (NF-AT) (65–67). Additionally, PKC activation leads to the inhibition of glycogen synthase kinase 3 beta (GSK-3β), which promotes the accumulation of beta-catenin in the nucleus and thus up-regulates the expression of c-Myc and cyclin D (68).

B cell scaffold protein with ankyrin repeats 1 (BANK1) interacts with PLCγ2, which is mediated by B-lymphocyte kinase (Blk) (69). BANK1 promotes the Lyn-mediated phosphorylation of the inositol trisphosphate receptor (IP3R) located on the endoplasmic reticulum which mediates Ca^2+^ flux (70).

### Negative Signaling Cascade

The role of Lyn in generating the positive signaling cascade in B cells is not essential and can be compensated for the other SFKs (71, 72). However, the role of Lyn in the initiation of negative feedback loops that not only regulate downstream signaling molecules but also limit the activity of SFKs is unique (35, 73). In addition to the phosphorylation of ITAMs after BCR stimulation, Lyn phosphorylates ITIMs contained within receptors including CD22, FcγRIIB, PIR-B, PD-1, CD66a (CEACAM1), CD5, and CD72 (35, 74–79), which act as docking sites for the binding and activation of non-receptor SH2-containing tyrosine phosphatases 1 and 2 (SHP-1 and SHP-2) and, in the case of FcγRIIB, phosphatidylinositol phosphatase 5 (SHIP-1) (76, 80–86). SHP-1 has been demonstrated to dephosphorylate and, therefore, down-regulate the activity of Btk, Syk, and Lyn, leading to the inhibition of signaling cascades (85, 87–89). Similarly, SHP-2 has been shown to dephosphorylate Ig-β, Syk, and PLCγ2 following BCR cross-linking (76). However, SHP-2 has also been implicated in enhancing various signaling pathways (90).

**Figure 2:**
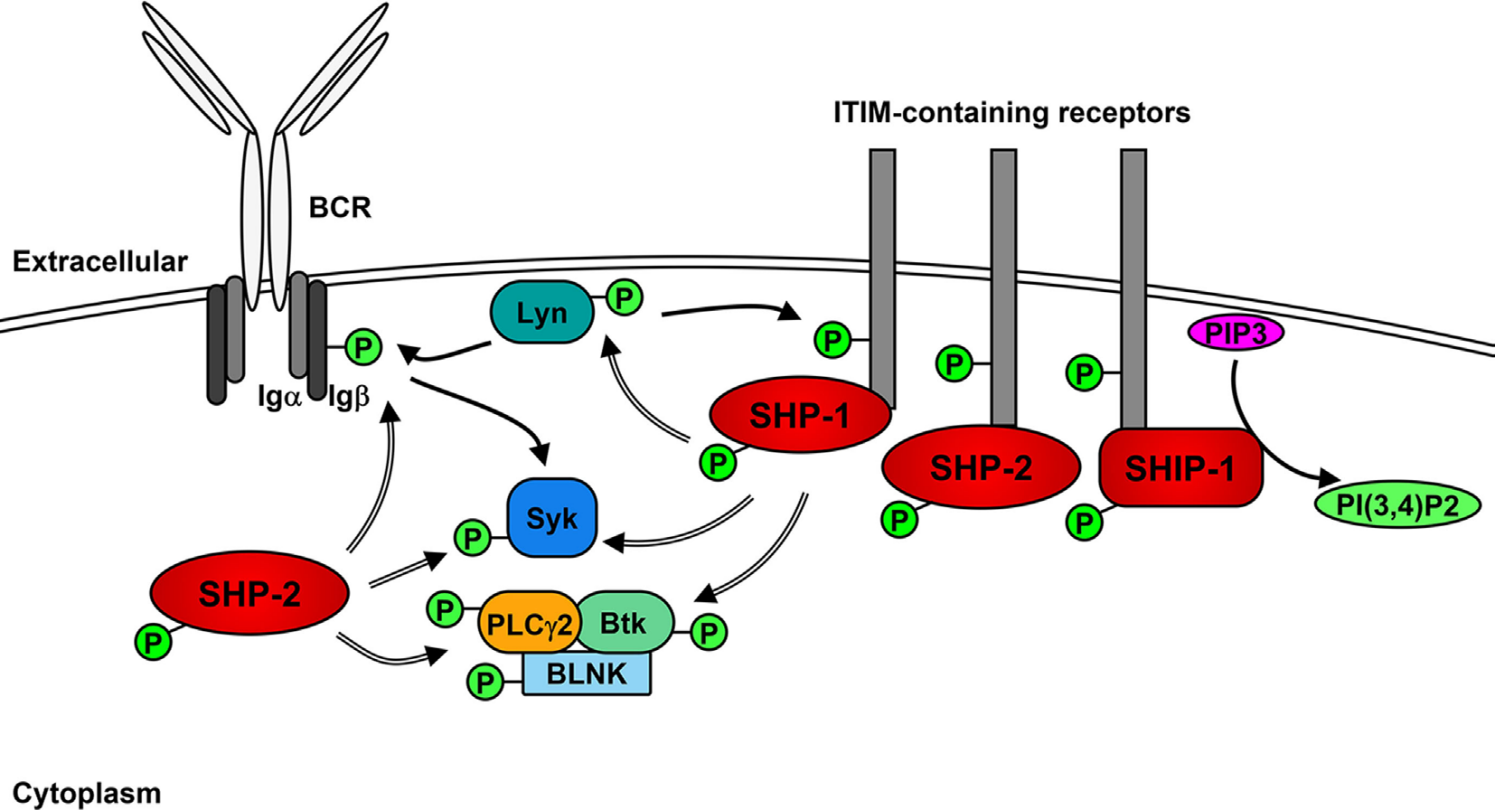
Lyn activates negative receptors following B cell receptor (BCR) cross-linking. In addition to initiating positive signaling, Lyn phosphorylates receptors containing immunoreceptor tyrosine inhibitory motifs (ITIMs), leading to the recruitment and activation of phosphatases SHP-1, SHP-2, and SHIP-1. SHP-1 and -2 dephosphorylate (=> arrows) and inhibit the activity of Lyn, Syk PLCγ2 and Btk leading to the down-regulation of positive signaling events. SHIP-1 dephosphorylates PIP3 and prevents activation of Akt signaling pathways.

Upon BCR activation (Figure 2), SHIP-1 dephosphorylates PIP3 to P(3,4)P2, which decreases PI3K- and Akt-mediated signaling (91). The decrease of available PIP3 also leads to the down-regulation of Btk activity and subsequent reduction of PLCγ2-mediated Ca^2+^ mobilization (92). Activated SHIP-1 recruits downstream of tyrosine kinase 1 (Dok1/p62dok), which down-regulates MAPK signaling pathways (93). FcγRIIB phosphorylation leads to the Dok3-mediated recruitment of SHIP-1 into the Grb2/Shc/mSOS complex, which inhibits SFK-dependent activation of Syk and decreases NF-κB and MAPK signaling pathways (94–97). A more direct role of Lyn in negative signaling is the phosphorylation of the Csk-binding protein/phosphoprotein associated with glyco-sphingolipid microdomains (Cbp/PAG), which activates Csk to down-regulate the activity of Lyn and other active SFKs (98).

Lyn also mediates internalization of the BCR, which acts to dampen signaling by the BCR (99) and may also play a role in the dephosphorylation of BCAP, which down-regulates PI3K activity (51). Similar to the regulation of active Src (100), the kinase activity of Lyn also promotes its own ubiquitination and subsequent degradation in B cells, most likely via the activity of Csk and suppressor of cytokine signaling (SOCS) proteins (101–104).

## *In Vivo* Models of Lyn Activity and Autoimmunity

Consistent with the important role for negative signaling in the hematopoietic compartment, mice lacking Lyn (Lyn^−/−^) progressively develop symptoms of autoimmunity that are comparable to SLE in humans (105). Lyn^−/−^ mice produce high titers of antinuclear antibodies (ANA) and develop splenomegaly, systemic inflammation, antibody complex deposition in the kidneys and glomerulonephritis (105). B cells from Lyn^−/−^ mice display hyper-phosphorylated Akt, MEK1/2, Erk1/2, and JNK compared to wild-type (WT) B cells after BCR stimulation, indicating enhanced positive signaling in the absence of negative feedback inhibition (23, 72, 106). In keeping with this, there is a reduced phosphorylation of FcγRIIB, CD22, SHP-1, and SHIP-1 (73, 74).

The activation of Lyn-dependent inhibitory signaling in mature B cells is essential for maintaining B cell tolerance (107), including B cell anergy (108). In Lyn-deficient mice, the absence of inhibitory signaling in mature B cells increases their sensitivity to antigen stimulation and this population of now auto-reactive cells is selectively depleted via clonal deletion (109). As such, Lyn^−/−^ mice show a significant reduction in naive, mature B cells in the periphery compared to WT mice despite similar frequencies of newly formed, immature B cells (105). The naive B cells that do persist in the periphery of Lyn^−/−^ mice are, however, hyper-responsive to anti-IgM stimulation, show delayed but increased Ca^2+^ mobilization and express markers of activation (107, 109–111). Additionally, antibody secreting B cells (plasma cells) persist in lymphoid tissues at 10-times normal frequency (105, 111). The myeloid compartment is also expanded, and T cells display markers of activation (101, 112). Increased serum levels of IL-6, IFN-γ, and BAFF, primarily produced by B cells, T cells, and myeloid cells respectively, promote the activation and proliferation of immune cells, further driving autoimmunity (112, 113).

Components of the autoimmune phenotype of Lyn^−/−^ mice are B cell intrinsic. This has been demonstrated using a B-cell-specific deletion of Lyn (Lyn^fl/fl^ mb-1Cre), which recapitulates the generation of hyper-responsive B cells, auto-antibodies and the development of glomerulonephritis (114). This phenotype may be dependent on signaling through toll-like receptor (TLR) adaptor protein myeloid differentiation primary response gene 88 (MyD88), as B-cell-specific deletion of MyD88 from Lyn^−/−^ B cells ameliorated auto-antibody production, T cell activation, myeloid expansion, and the development of glomerulonephritis (114). Additionally, global deletion of MyD88 from Lyn^−/−^ mice attenuated autoimmune disease development, which was considered to be due to reduced production of inflammatory cytokines IL-6 and IL-12 by Lyn^−/−^ dendritic cells (115, 116). This result was supported by Lyn being found to negatively regulate TLR-MyD88-IRF5-dependent expression of Type 1 IFNs in dendritic cells and by the double knock out of Lyn and IRF5 (Lyn^−/−^ IRF5^−/−^) in mice ameliorating SLE-like pathology (117). Perhaps surprisingly, the persistence of plasma cells was found to be independent of autoimmune disease and intrinsic to the Lyn^−/−^ hematopoietic compartment (118). Similarly, mice double deficient in Lyn and IL-6 retain the plasma cell hyperplasia but show a dramatic reduction in kidney damage, splenomegaly, and the production of ANAs (112). As in B cells, Lyn controls intracellular signaling intensities within plasma cells in response to stimulation by cytokines thought to play a critical role in plasma cell survival (118).

The deletion of MyD88 in Lyn^−/−^ mice also decreased spontaneous germinal center (GC) formation thus implicating GC reactions in the generation of pathogenic ANA in Lyn^−/−^ mice (119). In line with this, removing T cells (TCRβ/δ^−/−^) or deleting the adaptor protein SAP (SAP^−/−^), thereby preventing T cell B cell interactions required for GC formation, leads to the reduction of IgG auto-antibodies in Lyn^−/−^ mice (119). Similarly, deleting IL-21, a key regulator of GC responses and plasma cell formation (120), in Lyn^−/−^ mice leads to reduced IgG ANAs but does not alleviate IgM ANA, plasmacytosis, or glomerulonephritis (121), suggesting that IgM and/or IgA can mediate the development of autoimmunity (122). In keeping with this, the autoimmune disease in the *sanroque* lupus mouse model also develops in the absence of germinal centers and depends on IgM (123).

While Lyn can act as a positive or negative regulator of signaling cascades, its role as a negative regulator is critical to the development of the phenotype seen in Lyn^−/−^ mice. Indeed, the fact that dysregulation of other modulators of negative signaling, which are themselves targets of Lyn, also leads to autoimmunity (e.g., CD22, FcγRIIB, SHP-1, and SHIP-1) (124–128) helps define the pathways involved. This is also apparent in the compounding autoimmune disease phenotype in mice heterozygous in Lyn and SHP-1 (Lyn^+/−^, Mev^+/−^) (129). Finally, the importance of Lyn-regulated pathways in hematopoietic cells other than B cells is revealed by co-deletion of Btk, a key intermediate of several positive signaling pathways, from Lyn^−/−^ mice. This alleviates symptoms of autoimmunity and the production of auto-antibodies, but B cells remain hyper-responsive to anti-IgM stimulation as measured by Ca^2+^ mobilization and the phosphorylation of Erk1/2 and Akt (130, 131). Consistent with this, mice deficient in Lyn and p110δ, a PI3K isoform, also show a reduction in inflammation, splenomegaly, T cell activation, ANA production, and glomerulonephritis, while hyper-phosphorylation of Akt and Erk1/2 compared to control mice after BCR cross-linking remained (132), indicating a unique requirement for Lyn in regulating these signaling responses, but one that is insufficient by itself to permit development of disease.

Lyn’s enzymatic activity appears to be critical to its function, as mice expressing Lyn with no or impaired kinase activity still develop autoimmune disease, albeit with delayed onset and reduced severity (23, 24). B cells from Lyn^Mld4^ or kinase dead Lyn (Lyn^KD^) mice, harboring a mutation within the activation loop, display similar signaling kinetics to Lyn^−/−^ B cells, with hyper-Ca^2+^ mobilization, hyper-phosphorylated Erk1/2, Akt, and JNK and reduced phosphorylation of Syk, SHIP-1, and SHP-1 after BCR stimulation (23). Expansion of the myeloid compartment, splenomegaly, and the production of IgG anti-dsDNA antibodies were significantly reduced in Lyn^KD^ compared to Lyn^−/−^ mice, but evidence of immune complex deposition in the kidneys remained (23). In contrast are Lyn^WeeB^ mice, with a mutation in the G-loop of Lyn that leaves partial kinase activity, conferring B cell signaling kinetics that are intermediate between WT and Lyn^−/−^ (24). Stimulation of Lyn^WeeB^ B cells results in a partial decrease in the phosphorylation of Syk, Btk, PLCγ2, SHIP-1, and CD22 and a slight increase in the phosphorylation of Erk1/2, Akt, and JNK compared to WT B cells (24). However, in older Lyn^WeeB^ mice, splenomegaly, anti-dsDNA antibodies, and glomerulonephritis were comparable to those in Lyn^−/−^ mice (24), indicating that the partial positive signaling permitted by Lyn^WeeB^ requires greater negative signaling to counterbalance its activity and prevent the development of autoimmunity. This is exemplified in mice expressing a mutant form of constitutively active Lyn (Lyn^Y508F^ or Lyn^up/up^), as they develop an autoimmune disease with an increased rate of mortality (male-specific) compared to Lyn^−/−^ mice (102). Lyn^up/up^ B cells display constitutive phosphorylation of proteins involved in positive (Syk, PLCγ2) and negative (CD22, SHP-1, SHIP-1, FcγRIIB) signaling pathways, which is further increased after BCR stimulation (102). Despite the increased phosphorylation of ITIM-containing negative regulators, Lyn^up/up^ B cells display enhanced Ca^2+^ mobilization compared even to Lyn^−/−^ B cells, indicating that the increased positive signaling further outweighs the inhibitory signaling capacity within these cells (102). Thus, in the hematopoietic compartment, Lyn activity controls both negative and positive signaling and its dysregulation in mice is responsible for the breakdown of tolerance in B cells and progressive development of autoimmunity.

## Lyn and SLE

Systemic lupus erythematosus is a heterogeneous disease with varied clinical presentations and manifestations (1). It is therefore not surprising that its cause is multifactorial, with numerous genetic and environmental factors contributing to pathogenesis (1, 133). As such, a single gene defect in mice such as Lyn^−/−^ is unlikely to replicate the complex phenotype seen within SLE in humans. Despite this, a reduction in Lyn expression, via increased turnover or reduced transcription, and altered sub-cellular localization are reported in patients with SLE (134–136). However, a significant susceptibility association of the *Lyn* locus with SLE has only been determined in a single case–control association study (137). Interestingly, proteins involved in the positive (Blk, BANK1) and negative (PTPN22/PEP, Csk, FcγRIIA, FcγRIIB, FcγRIIIA, FcγRIIIB, SOCS1) signaling pathways in B cells have all been linked to SLE via genome-wide association studies (GWAS) and this highlights the importance of regulating these signaling cascades to avoid disease onset or progression (138–143). Similarly, Blimp1 and Ets1, transcription factors involved in regulating plasma cell development, are also linked with SLE (141, 144). Mice deficient in *Ets1* show enhanced generation of plasma cells and auto-antibodies, which are symptoms of autoimmunity (145). Recently, Lyn was linked with regulating *Ets1* expression, via the activation of CD22 and SHP-1 reducing the Btk-dependent down-regulation of *Ets1* (146, 147).

Lyn^−/−^ mice are one of the numerous mouse models reported to develop an SLE-like illness, with none being a perfect recapitulation of the spectrum of human disease (148). Regardless, the Lyn^−/−^ mouse model has been and remains a useful tool in dissecting the critical role that B cell signaling pathways play in the development of autoimmunity. Indeed, other models in conjunction with Lyn^−/−^ mice have confirmed the importance of B cell dysregulation to the development of SLE-like autoimmunity. Deleting B cells in the MRL/lpr mouse model, for example, significantly decreases disease progression and mortality compared to mice that remain B cell replete (149). Furthermore, comparison of Lyn^−/−^ with other mouse models has identified several potential novel therapies for patients with SLE such as inhibitors of BTK and HDAC (150, 151). Further determination of the molecular pathways responsible for B cell dysregulation in SLE-like autoimmunity will likely assist in the design of new treatments to ameliorate disease severity.

## Concluding Remarks

The role of Lyn in B cells involves fine-tuning of BCR signaling, balancing positive and negative signals to maintain tolerance to self-antigens while permitting responsiveness to foreign antigens. The Lyn-mediated phosphorylation of ITIM-containing negative receptors and subsequent activation of the inhibitory phosphatases, SHP-1 and SHIP-1, that leads to the down-regulation of BCR-mediated signaling cascades and inhibition of SFK activity represents a critical component in B cell signaling that prevents the development of autoimmunity.

Although defects in Lyn^−/−^ mice are not an identical model for human SLE, the investigation of Lyn and the pathways it modulates have highlighted the delicate balance inherent in B cell kinase signaling cascades and the devastating consequences that can occur when they are dysregulated. Numerous SLE susceptibility genes identified through GWAS are also linked with other autoimmune diseases indicating the involvement of shared pathways that ultimately lead to the loss of tolerance (152). Therefore, future experiments examining genomic regulation or global phospho-proteomics in models of SLE could be useful in identifying all the components of the intracellular pathways involved and through that, potential therapeutic targets.

## Author Contributions

EB, SI, and ML drafted the article. DT contributed to writing and provided critical review. All authors were involved in the revising process and approve the manuscript for submission.

## Conflict of Interest Statement

The authors declare that the research was conducted in the absence of any commercial or financial relationships that could be construed as a potential conflict of interest. The handling Editor declared a shared affiliation, though no other collaboration, with one of the authors [ML].
